# Defining COVID-19 as a Disaster Helps Guide Public Mental Health Policy

**DOI:** 10.1017/dmp.2020.301

**Published:** 2020-08-12

**Authors:** Abdulaziz Alkhayyat, Kishan Pankhania

**Affiliations:** Faculty of Biology, Medicine and Health, University of Manchester, Oxford Road, Manchester, UK

**Keywords:** public health, public policy, psychological stress, disasters, pandemics, public mental health

## Abstract

The coronavirus disease (COVID-19) pandemic continues to be a devastating chapter in history. The consequences of the pandemic unfold daily and they extend beyond physical health. Current research suggests that it is a public mental health crisis. With regard to the physical effects of COVID-19, policy-makers have drawn from past experiences, such as the severe acute respiratory syndrome (SARS) outbreak of 2003, to craft unique responses. A similar approach must be taken to address the mental health effects of the pandemic. Because COVID-19 can fit the definitions of a mental health disaster, it can be addressed using the principles of disaster mental health management. This letter to the editor presents arguments for defining COVID-19 as a mental health disaster, the challenges facing policy-makers in addressing it as such, and calls upon researchers to fill this gap in the literature.

The coronavirus disease (COVID-19) pandemic has been devastating. Furthermore, the havoc wreaked by the pandemic extends beyond physical health. Arguably, the pandemic is a mental health crisis. The literature surrounding COVID-19 supports this claim. In the work of Qiu et al.,^1^ a nationwide survey found that 35% of 52 730 respondents across China had experienced psychological distress associated with the pandemic. The grim uniqueness of COVID-19 is that the panic and fear that come with the virus extend beyond those infected.

To address the physiological ramifications of this pandemic, policy-makers are being tasked with creating unique public health policies. They are looking to past, similar events that serve as guides on how to respond. Hence, social distancing and quarantine measures were implemented because of their efficacy in past crises, such as the severe acute respiratory syndrome (SARS) outbreak in 2003.^2^ Yet, where do they turn to address the mental health effects of COVID-19? One may draw parallels between the pandemic and other catastrophic events, such as mass shootings, which inflicted significant trauma on communities.^3^ Thus, the answer to the psychological challenges posed by COVID-19 may lie in disaster mental health management.

Disaster mental health management takes a preventive approach in aiding people struck with disaster.^4^ Math et al. defines a disaster, in terms of mental health, as an event that meets the following criteria – sudden onset, unpredictability, uncontrollability, huge magnitude of destruction, human loss and suffering – and greatly exceed[s] the coping capacity of the affected community.^4^ The COVID-19 pandemic meets the aforementioned criteria and, therefore, could be deemed a disaster.

Henceforth, the psychological effects of COVID-19 should be addressed using the principles of disaster mental health management. This could include offering community-based services that administer psychological first aid, debriefing, and cognitive behavioral therapy among other interventions.^4^ This recommendation echoes the work of Qiu et al.^1^ who recommend “nationwide strategic planning and coordination for psychological first aid during major disasters, potentially delivered through telemedicine.” The challenge that we face in implementing these services is scaling them nationally and internationally to different health care systems. The authors anticipate that, if this approach is taken, the ill mental health effects of the pandemic will be significantly reduced. This, in effect, can decrease the burden placed on mental health services after the pandemic. Researchers should study this speculation and substantiate it further.

The consequences of not addressing COVID-19 as a mental health disaster could be significant. In disaster-stricken communities, mild and moderate mental disorders tend to double after the disaster.^5^ To avoid such repercussions associated with COVID-19, a considerable public health response must be urgently taken – one which views the pandemic as a disaster. Thus, researchers should conduct original research on COVID-19 through the lens of a disaster and seek means to avoid a mental health crisis. In other words, it is time to “flatten the curve” of the psychological effects of this pandemic.
